# Increased Autoreactivity of the Complement-Activating Molecule Mannan-Binding Lectin in a Type 1 Diabetes Model

**DOI:** 10.1155/2016/1825738

**Published:** 2016-02-10

**Authors:** Jakob Appel Østergaard, Marieta Milkova Ruseva, Talat Habib Malik, Ingeborg Torp Hoffmann-Petersen, Matthew Caleb Pickering, Steffen Thiel, Troels Krarup Hansen

**Affiliations:** ^1^Department of Endocrinology and Internal Medicine, Aarhus University Hospital and Department of Clinical Medicine, Faculty of Health, Aarhus University, 8000 Aarhus, Denmark; ^2^The Danish Diabetes Academy, 5000 Odense, Denmark; ^3^Centre for Complement and Inflammation Research, Imperial College London, London W12 0NN, UK; ^4^Department of Biomedicine, Faculty of Health, Aarhus University, 8000 Aarhus, Denmark

## Abstract

*Background*. Diabetic kidney disease is the leading cause of end-stage renal failure despite intensive treatment of modifiable risk factors. Identification of new drug targets is therefore of paramount importance. The complement system is emerging as a potential new target. The lectin pathway of the complement system, initiated by the carbohydrate-recognition molecule mannan-binding lectin (MBL), is linked to poor kidney prognosis in diabetes. We hypothesized that MBL activates complement upon binding within the diabetic glomerulus.* Methods*. We investigated this by comparing complement deposition and activation in kidneys from streptozotocin-induced diabetic mice and healthy control mice.* Results*. After 20 weeks of diabetes, glomerular deposition of MBL was significantly increased. Diabetic animals had 2.0-fold higher (95% CI 1.6–2.5) immunofluorescence intensity from anti-MBL antibodies compared with controls (*P* < 0.001). Diabetes and control groups did not differ in glomerular immunofluorescence intensity obtained by antibodies against complement factors C4, C3, and C9. However, the circulating complement activation product C3a was increased in diabetes as compared to control mice (*P* = 0.04).* Conclusion*. 20 weeks of diabetes increased MBL autoreactivity in the kidney and circulating C3a concentration. Together with previous findings, these results indicate direct effects of MBL within the kidney in diabetes.

## 1. Introduction

Diabetic kidney disease affects about 30% of patients with diabetes, causing impaired quality of life and putting significant strain on health care systems worldwide [1]. Despite intensive treatment of risk factors such as blood glucose and blood pressure, diabetic nephropathy remains the primary cause of end-stage renal failure which requires transplantation or dialysis [2, 3]. Persistent research to identify novel therapeutic targets is therefore of paramount importance.

The lectin pathway of complement activation is emerging as a potential drug target. Clinical observations through the last decade link the complement-activating carbohydrate-recognizing molecule mannan-binding lectin (MBL) to diabetic kidney disease [4–9]. In addition, we have recently described that the concentration-determining MBL genotype is tightly associated with mortality in type 1 diabetes patients [10]. Animal studies indicate a causal role of MBL in this association as MBL knockout mice are resistant to diabetic kidney changes [11, 12]. In vitro studies suggest that diabetes-induced glycoprotein alterations may adversely activate complement through binding of MBL to neoepitopes [13, 14]. These diabetes-induced neoepitopes may develop through either altered enzymatic protein glycosylation or nonenzymatic formation of advanced glycation end-products [15–17]. By use of plant lectins selective for specific carbohydrate patterns, it has been shown that diabetes alters the carbohydrate composition in tissues [18, 19]. Furthermore, diabetic kidney tissue has increased reactivity with the lectin concanavalin A which binds mannose [20]. Diabetes-induced deposition of complement factor C3 as a sign of complement attack has been demonstrated in rats [21–23]. In addition, complement regulatory proteins are inhibited by nonenzymatic glycation in diabetes which leads to accelerated complement attack [24, 25]. The ensuing sublytic effects induce a mitogen stimulus and growth factor release, which further links the complement system mechanistically to diabetic kidney changes [26, 27].

The specific contribution of the lectin pathway to the complement activation remains unknown, and no studies have investigated the autoreactivity of MBL in the kidney in diabetes. We hypothesize that MBL is deposited within the diabetic glomerulus as a consequence of binding to hyperglycemia-induced neoepitopes and that this initiates complement activation contributing to diabetic kidney damage. To test this hypothesis, we examined MBL deposition within the diabetic glomerulus and measured glomerular and circulating complement components.

## 2. Materials and Methods

### 2.1. Animals

Six-week-old C57BL/6JBomTac male mice (Taconic, Ry, Denmark) were randomized to a control group (*n* = 12) or a diabetes group (*n* = 20). At eight weeks of age, experimental type 1 diabetes was induced by streptozotocin. For five consecutive days, the animals in both groups were fasted for four to six h prior to injection with either the empty buffer in control group or low-dose streptozotocin (55 mg/kg, catalogue number (Ca) S0130 Sigma-Aldrich, St. Louis, MO, USA) dissolved in cold 10 mmol/L citrate acid buffer, pH 4.5, in the diabetes group [28]. For the remaining time, all animals had free access to standard chow (Altromin 1324, Lage, Germany) and tap water. Animals were housed as litter mates, two to eight per cage, in a room with an artificial light circle (light 6.00 a.m. to 6.00 p.m.), temperature at 21 ± 1°C, and 55 ± 5% humidity. The study complied with the Danish regulations for care and use of laboratory animals (license number 2012-15-2934-00113 of July 5, 2012).

Blood glucose was monitored throughout the study on tail-vein blood by Contour*™* (Bayer Diabetes Care, Kongens Lyngby, Denmark). If blood glucose was above the limit of quantification (33.3 mmol/L), the missing value was replaced by 33.4 mmol/L in statistical evaluations.

During the study and prior to quantification of any outcome measurements, seven animals in the diabetes groups were excluded (one animal did not develop diabetes after streptozotocin, two animals died, and four animals were sacrificed for ethical reasons because of severe weight loss).

### 2.2. Urinary Albumin Excretion Rate

One week before termination of the study, the animals were placed individually in metabolic cages for collection of urine. To lower physical stress to the animals during isolation, we only collected urine during a 16 h period from which 24 h urine production was estimated. Urinary albumin concentration was measured by Mouse Albumin ELISA Quantification Kit (Ca E90-134, Bethyl Laboratories, Inc., Montgomery, TX, USA).

### 2.3. Samples

Blood samples were drawn to EDTA-coated tubes (Ca 16.444.100, Sarstedt, Nümbrecht, Germany) at the start of study before induction of diabetes without anesthetizing the animals. At the end of study, the animals were anesthetized by intraperitoneal injection of ketamine and xylazine (0.5 mg/g body weight ketamine, 0.2 mg/g body weight xylazine, both from Intervet, Skovlunde, Denmark). Blood samples were drawn into EDTA-coated tubes and placed on wet ice. To avoid ex vivo complement activation and generation of complement-cleavage product C3a, we added Futhan immediately after drawing the end-of-study samples in accordance with manufacture's instruction (Ca 552035, BD Pharming, Oxford, UK). Plasma was separated after spin at 4°C and subsequently frozen to −80°C.

Dissected kidneys were immediately embedded in Tissue-Tek optimal cutting temperature (OCT, Ca 62550-01, Electron Microscopy Sciences, Hatfield, PA, USA) and frozen to −80°C. Poles of contralateral kidney were snap-frozen in liquid nitrogen for later RNA isolation.

### 2.4. Plasma C3 Profile

Circulating C3 concentration was quantified using Complement C3 Mouse ELISA kit according to manufacturer's instructions (Ca ab157711, Abcam, Cambridge, UK).

Circulating C3a concentration was determined by ELISA as previously described [29]. For capture, we used a rat anti-mouse antibody with specificity for the neoepitope generated by the cleavage of C3 and not recognizing intact C3 (Ca 558250, BD Pharming, Oxford, UK). Plates were coated with antibody diluted to 2 *μ*g/mL in borate buffered saline followed by overnight incubation at 4°C and subsequently blocked with sample buffer (PBS, 2% BSA, 0.1% tween-20, 0.02% NaN_3_). Plasma was incubated overnight at 4°C in sample buffer. Purified mouse C3a protein was used as standard (Ca 558618, BD Pharming, Oxford, UK). For detection we used biotinylated rat anti-mouse C3a antibodies (0.5 *μ*g/mL in sample buffer, Ca 558251, BD Pharming, Oxford, UK) which was incubated for 5 h at 4°C. This was followed by addition of streptavidin-alkaline phosphatase and later development with alkaline phosphatase substrate.

### 2.5. Immunofluorescence

Five-*μ*m thick cryosections were cut from the OCT embedded kidney blocks and fixed in acetone. Complement factors were visualized with the following antibodies: biotinylated rat anti-mouse MBL-C [30], FITC-conjugated goat anti-mouse C3 (Ca 55500, MP Biomedicals, Illkirch Cedex, France), FITC-conjugated rat anti-mouse C4 (Ca CL7504F, Cedarlane, Burlington, NC, USA), and rabbit anti-rat C9 antibodies cross-reacting with mouse C9 (gift from B. Paul Morgan, Cardiff University, UK). Mouse IgG was stained with FITC-conjugated goat anti-mouse IgG antibody (Ca F5383, Sigma-Aldrich, St. Louis, MO, USA). The biotinylated antibodies against MBL-C were visualized by streptavidin-conjugated Alexa 488. Rabbit anti-C9 antibodies were visualized by Alexa Fluor 594-conjugated F(ab′)2 of goat anti-rabbit IgG polyclonal antibody (Ca A11072, Life Technologies, Paisley, UK). Complement staining intensity was quantified in Image Pro Plus software (MediaCybernetics, Rockville, MD, USA) as the median immunofluorescence from 10–20 random glomerular profiles measured at ×400 magnification using an Olympus BX4 fluorescence microscope (Olympus 6821 Optical, London, United Kingdom) with a Photonic Science Color Coolview digital camera attached (Photonic Science, East Sussex, UK) [31].

### 2.6. PCR

Glomeruli from snap-frozen kidney poles were isolated in sterile PBS by the standard sieving technique as previously described [32]. RNA was extracted by PicoPure RNA isolation kit (Ca KIT0204). Complementary DNA was generated by High Capacity cDNA Transcription Kit (Ca 4368814). Real-time PCR was performed with TagMan MasterMix (Ca 4364338) containing target* Mbl2* primer (Ca Mm00487623_m1) and housekeeping gene 18S primer (Ca 4319413E) all from Life Technologies, Paisley, UK. Liver tissue was used as positive control.

### 2.7. Statistics

For normally distributed data, the groups were compared by Student's *t*-test and estimates were expressed as mean (95% CI). If normal distribution could not be obtained by transformation of data by natural logarithm, Wilcoxon rank-sum test was used and the estimates were expressed as median (interquartile range (IQR)). In graphical illustration of data, the within-group median is indicated for all outcomes for consistency. The alpha level for two-sided tests was set to 5%. As described above, blood glucose concentrations above the limit of quantification (33.3 mmol/L) were set to the value 33.4 mmol/L used in statistical evaluation.

## 3. Results and Discussion

### 3.1. Results

#### 3.1.1. Blood Glucose and Body Weight

Blood glucose levels of the animals randomized to the diabetes group and the control group did not differ before the first streptozotocin injection (7.1 mmol/L [IQR 6.6–7.3] in diabetes group versus 6.8 mmol/L [IQR 6.4–7.8] in the control group, *P* = 0.82). All animals were sacrificed 20 weeks after diabetes was established. At sacrifice, the diabetes group had significantly higher blood glucose compared with the control group (33.4 mmol/L [IQR 28.6–33.4] in the diabetes group versus 7.1 mmol/L [IQR 6.0–7.9] in the control group, *P* < 0.001).

Animals in the two groups did not differ in body weight before first streptozotocin injection (24.4 g [95% CI 23.4–25.3] in the diabetes group versus 24.0 g [95% CI 22.9–25.1] in the control group, *P* = 0.54). At the end of the study, body weight of the diabetic animals (21.1 g [95% CI 19.7–22.3]) was significantly lower than the control animals (35.8 g [95% CI 33.5–38.1]), *P* < 0.001.

#### 3.1.2. Kidney-to-Body Weight Ratio

**Figure 1:**
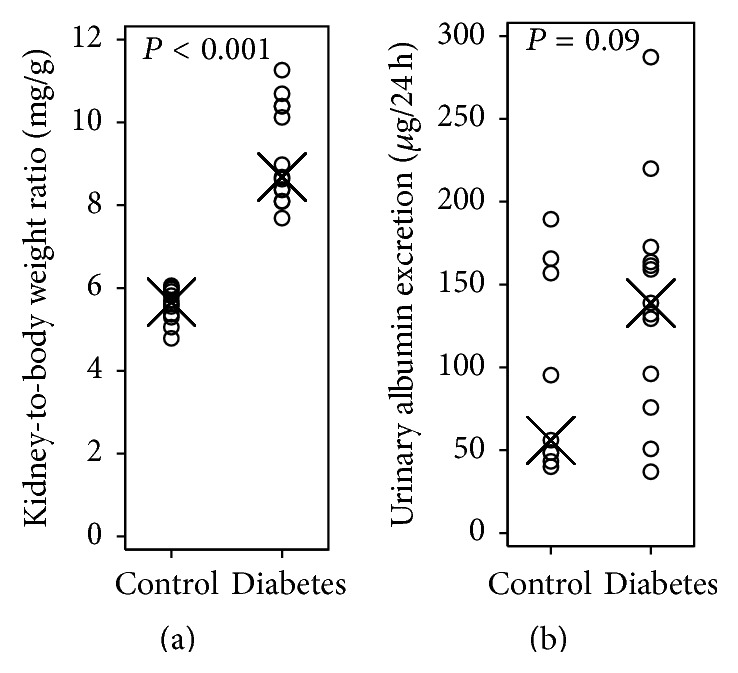
(a) Kidney weight normalized to body weight in control and diabetes group. Within-group median is indicated with X. *P* value refers to test statistics from *t*-test. (b) Estimated 24 h urinary albumin excretion in control and diabetes group. Within-group median is indicated with X. *P* value refers to test statistics from *t*-test on normally distributed, logarithmic-transformed data.

Kidney weight was normalized to final body weight of the animal to adjust for body weight differences between the two groups. After 20 weeks of diabetes, the relative kidney weight was markedly increased, as expected, in the diabetes group as compared with controls (Figure 1(a)). The kidney-to-body weight ratio was 9.2 mg/g (95% CI 8.5–9.9) in diabetic mice as compared with 5.6 mg/g (95% CI 5.3–5.9) in age-matched control mice, *P* < 0.001.

#### 3.1.3. Urinary Albumin Excretion (UAE) Rate

No difference in urinary albumin excretion was found between the groups (Figure 1(b)). Without reaching statistical significance at the 0.05-level, the diabetic animals had a 1.6-fold (95% CI 0.9–2.7) higher UAE than control animals, *P* = 0.09.

#### 3.1.4. Glomerulus Immunofluorescence

**Figure 2:**
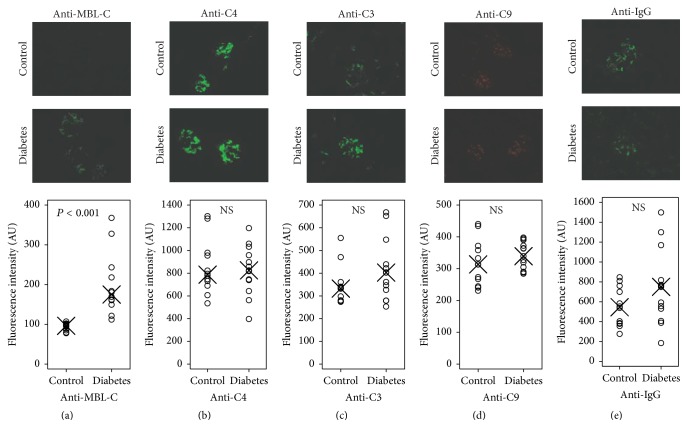
Illustrative pictures of glomerular staining of complement factors and dot plots of immunofluorescence quantification. Within-group median of immunofluorescence measurements is indicated with X. (a) Anti-MBL-C stain; *P* value refers to test statistics from *t*-test on normally distributed, logarithmic-transformed data. (b) Anti-C4 stain; NS refers to nonsignificant test statistics in *t*-test. (c) Anti-C3 stain; NS refers to nonsignificant test statistics in *t*-test. (d) Anti-C9 stain; NS refers to nonsignificant test statistics in *t*-test. (e) Anti-IgG stain; NS refers to nonsignificant test statistics in *t*-test.

The staining for MBL-C was intense in the diabetic animals as opposed to almost no staining in controls (Figure 2(a)). The quantified glomerular MBL fluorescence intensity was 2.0-fold (95% CI 1.6–2.5) higher in diabetes as compared with control, *P* < 0.001.

We subsequently stained for C4 which is deposited (in the form of C4b) upon complement activation through the lectin and classical pathway. In both groups, we observed bright staining but no difference in intensity between the groups, *P* = 0.77 (Figure 2(b)).

Following deposition of C4b, complement factor C2 binds to C4b and is transformed to a C3-activating enzyme (by MBL-associated serine protease-2 in the lectin pathway or by C1s in the classical pathway), leading to deposition of C3b at the site of activation. When examining C3 deposition, we did not find any difference between the two groups, *P* = 0.19 (Figure 2(c)).

The final step of complement attack on a cell surface is the incorporation of complement factor C9 into the membrane and formation of the membrane-attack complex (C5b-9). We did not find any statistical difference in the fluorescence intensity from anti-C9 antibodies when comparing diabetic and control animals, *P* = 0.34 (Figure 2(d)).

As control we stained for IgG which did not differ between the two groups, *P* = 0.23 (Figure 2(e)).

We planned to quantify glomerular* Mbl2 *transcription to evaluate whether the diabetes-induced increased anti-MBL-C staining was caused by deposition of circulating MBL or by increased local MBL production. However, although the* Mbl2* transcript was too low for quantification, the total RNA yield from isolated glomeruli was very low and we were therefore unable to make any reliable conclusions from transcript analysis. The housekeeping gene did give positive signal in isolated glomeruli. In liver positive controls, we observed signal from both* Mbl2* and housekeeping gene.

#### 3.1.5. Plasma C3 Profile

**Figure 3:**
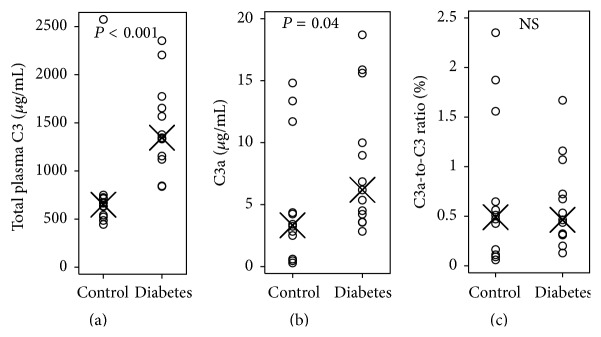
Plasma concentration of C3 in control and diabetic group with the within-group median indicated with X. (a) Total C3 concentration; *P* value refers to Wilcoxon rank-sum test. (b) Complement activation product C3a concentration; *P* value refers to Wilcoxon rank-sum test. (c) Ratio between plasma C3a and total plasma C3; NS refers to nonsignificant Wilcoxon rank-sum test.

Total circulating C3 concentration was determined in plasma samples drawn at sacrifice (Figure 3(a)). We found the C3 concentration to be significantly higher in diabetic animals (1375 *μ*g/mL [IQR 1156–1654]) compared with control animals (651 *μ*g/mL [IQR 532–719], *P* < 0.001). The concentration did not differ at baseline (data not shown).

To estimate systemic complement activation, we measured circulating C3a concentration which is generated when C3 is cleaved to C3b and C3a and released to circulation. At the end of the study, the C3a concentration was significantly higher in diabetic animals (6.2 *μ*g/mL [IQR 4.2–10.0]) compared with controls (3.3 *μ*g/mL [IQR 1.6–8.0]), *P* = 0.04 (Figure 3(b)). No difference between the groups was found at baseline (data not shown).

As the total C3 concentration was increased in diabetes, we calculated the ratio between activated C3a and total C3 concentration as measure of relative C3 activation. Even though the total concentration of activated C3a was higher in diabetes, the relative C3 activation did not differ between the two groups as estimated from the C3a-to-C3 ratio (Figure 3(c), *P* = 0.83).

### 3.2. Discussion

The present results support our hypothesis as autoreactivity of MBL towards kidney tissue is increased in diabetes. In addition, we find increased complement activation at systemic level by measurement of plasma C3a. The proposed mechanism is that prolonged hyperglycemia in diabetes leads to glycoprotein alterations which may enable adverse complement activation through binding of MBL to neoepitopes. However, we were not able to detect increased complement activation in diabetic glomeruli, measured as immunofluorescence of C4, C3, or C9.

High MBL concentration is associated with diabetic kidney disease in several clinical cohorts [4–9]. A causal role of MBL in diabetic kidney disease is indicated in animal studies showing that MBL deficiency attenuates diabetes-induced kidney changes, but the underlying mechanisms have not been identified [11, 12].

MBL is a pattern-recognition molecule that binds specific carbohydrate patterns [33]. These carbohydrate patterns are commonly found on yeast and bacteria in sufficient density to allow high-avidity binding of multiple subunits in the MBL polymer. Normally, MBL does not activate complement in healthy tissue as normal human glycoproteins are not recognized with a sufficiently high binding strength. Furthermore, a large number of complement-regulating proteins in circulation and on cell surfaces minimize complement self-attack in humans. In addition to the antimicrobial activity, MBL also plays a role in tissue homeostasis, for example, by opsonizing apoptotic cells [34, 35].

A proposed mechanism is hyperglycemia-driven alterations in carbohydrate patterns that enable MBL-mediated adverse complement self-attack in diabetes [6, 13]. In vitro studies suggest that MBL is able to recognize advanced glycation end-products and activate complement, but whether this resembles in vivo conditions is not known [13, 14].

The present study therefore aimed to investigate three questions: firstly, if MBL autoreactivity is increased within the diabetic glomerulus; secondly, if glomerular complement activation is increased; and thirdly, if diabetes increases the degree of complement activation at systemic level.

The present study shows that diabetic animals have a more intense glomerular staining for MBL as compared with nondiabetic controls. As the local* Mbl2* transcription was below the limit of quantification, we conclude that the MBL staining arises from MBL deposition from the circulating pool. These results point towards a direct effect of MBL in diabetic kidney disease.

The next question we asked was if the increased MBL deposition would translate into an increased staining for the downstream complement components. These complement components are gradually deposited on the target during successive steps of complement factor cleavages. We therefore stained for C4, C3, and C9 in order to detect complement activation within the kidney.

We found positive staining for all the downstream complement factors, but we were not able to detect any differences between the diabetic and nondiabetic groups. This might be explained by insufficient sensitivity to detect small differences by immunofluorescence. Diabetes complications in the kidney normally develop slowly over a long time span, and even very small degrees of complement activation, undetectable by immunofluorescence, may eventually lead to an accelerated development of kidney disease. Immunofluorescence as method thus limits the ability of our study to detect subtle differences in complement activation that might prove to be of physiological relevance. As complement is activated ex vivo as function of time and temperature, it is not possible to use existing human samples collected without control of complement for post hoc evaluation of diabetes-induced complement activation [36]. Thirdly, we measured complement activation at the systemic level by quantifying the cleavage product C3a in plasma in an assay not recognizing the nonactivated intact C3. Plasma samples from the end of the study were collected with admixture of an enzyme inhibitor and rigorous control to avoid spontaneous ex vivo complement activation [36]. We found that diabetic mice had increased C3a concentrations as compared with control mice indicating a higher degree of complement activation. The plasma concentration of total C3 (including nonactivated C3) was also increased in diabetes. While C3 is predominantly produced in the liver, C3 has been reported to be transcribed and translated at a higher level within glomeruli of patients with diabetic kidney disease as compared with healthy controls [37]. Low RNA yield in sieve-isolation of glomeruli prevents our study from similar assessments. Although total C3 and activated C3a were measured in two different assays on different standard curves, we calculated the relative C3 activation as the ratio between C3a and total C3 concentrations. From this ratio we find that while the absolute complement activation measured by circulating C3a is higher in diabetes, the relative activation did not differ between groups.

## 4. Conclusions

In conclusion, the present study shows that diabetes leads to MBL deposition within the glomerulus and a higher plasma concentration of the complement activation product C3a. This indicates that the previously described role of MBL in diabetic kidney disease can be explained by MBL's recognition of hyperglycemia-induced neoepitopes in the diabetic glomerulus. From the increase in circulating C3a concentration in diabetes, it is reasonable to speculate that complement may also be activated outside the kidney at more abundant sites, for example, the vascular wall. However, this needs to be investigated in future studies.
